# Association of 25-hydroxyvitamin D and chemerin with metabolic syndrome components in individuals with type 2 diabetes mellitus

**DOI:** 10.3389/fmed.2026.1907839

**Published:** 2026-08-07

**Authors:** Mukhtiar Baig, Zohair Jamil Gazzaz, Mohamad Nidal Khabaz, Shahad Abduljalil Abualhamael, Sami Hamdan Alzahrani, Wail A. Alamoudi

**Affiliations:** 1 Department of Clinical Biochemistry and Medical Education, Faculty of Medicine, King Abdulaziz University, Jeddah, Saudi Arabia; 2 Department of Internal Medicine, Faculty of Medicine, King Abdulaziz University, Rabigh, Saudi Arabia; 3 Department of Pathology, Faculty of Medicine, King Abdulaziz University, Rabigh, Saudi Arabia; 4 Department of Family Medicine, Faculty of Medicine, King Abdulaziz University, Jeddah, Saudi Arabia; 5 Health Promotion Center, King Abdulaziz University, Jeddah, Saudi Arabia; 6 Family Medicine and Chronic Diseases Research Unit, King Fahd Medical Research Center, King Abdulaziz University, Jeddah, Saudi Arabia

**Keywords:** 25-hydroxyvitamin D, chemerin, CRP, dyslipidemia, inflammation, insulin resistance, metabolic syndrome, T2DM

## Abstract

**Background:**

This study investigated the associations of serum 25-hydroxyvitamin D [25(OH)D], the principal circulating marker used to assess vitamin D status, and chemerin with metabolic syndrome (MetS) components in individuals with type 2 diabetes mellitus (T2DM) and in a non-diabetic comparison group. The MetS components assessed were central obesity/waist circumference, blood pressure, fasting plasma glucose, triglycerides, and HDL-C, because these are the diagnostic elements of the International Diabetes Federation (IDF) definition.

**Methods:**

This cross-sectional analytical study was conducted at King Abdulaziz University, Rabigh, between August 2021 and December 2022. Participants were classified as having MetS according to IDF criteria, and vitamin D status was classified using serum 25(OH)D concentrations as deficient, insufficient, or sufficient. Serum 25(OH)D, chemerin, and CRP were measured by ELISA.

**Results:**

A total of 176 participants were included in the study (88 with diabetes and 88 non-diabetic comparison participants), with equal sex distribution in both groups (44 females and 44 males per group). Compared with the non-diabetic comparison group, participants with T2DM had significantly higher chemerin (*p* < 0.001) and significantly lower 25(OH)D (*p* < 0.001). The MetS was more common in diabetic participants than in non-diabetic comparison participants: 70/88 (79.5%) vs. 43/88 (48.9%) (*p* < 0.001). Blood chemerin levels were also elevated in those with MetS compared with those without MetS (*p* = 0.032). Within the diabetic group, 18/88 (20.5%) had 25(OH)D deficiency and 47/88 (53.4%) had insufficient levels, whereas among non-diabetic comparison participants, 7/88 (8%) had deficiency and 32/88 (36.4%) had insufficient levels (*p* < 0.001). Higher FPG, insulin, HOMA-IR, HbA1c, TC, and CRP were associated with lower 25(OH)D in diabetic subjects. The latter inverse relationships were also observed in the MetS subgroup. Chemerin was associated with HOMA-IR, FPG, TC, CRP, and HbA1c in diabetic subjects. In the MetS subgroup, FPG, insulin, HOMA-IR, HbA1c, TC, and CRP remained positively correlated with chemerin. The regression analysis revealed that diabetic status and female sex were independently related to 25(OH)D status. In the adjusted chemerin model, chemerin was inversely and significantly associated with 25(OH)D (B −2.451, 95% CI −3.916–−0.986, *p* = 0.001). Diabetes and female sex were the only variables independently associated with MetS status in the logistic regression analysis.

**Conclusion:**

Individuals with T2DM showed lower serum 25(OH)D and increased chemerin concentrations than non-diabetic comparison participants. Chemerin was higher in participants with MetS, whereas neither 25(OH)D nor chemerin was independently associated with MetS in the adjusted logistic model. These findings support an association, rather than a causal or diagnostic relationship, between 25(OH)D, chemerin, and cardiometabolic disturbance in this cohort. Further longitudinal and interventional studies are required.

## Graphical Abstract

**Figure:**
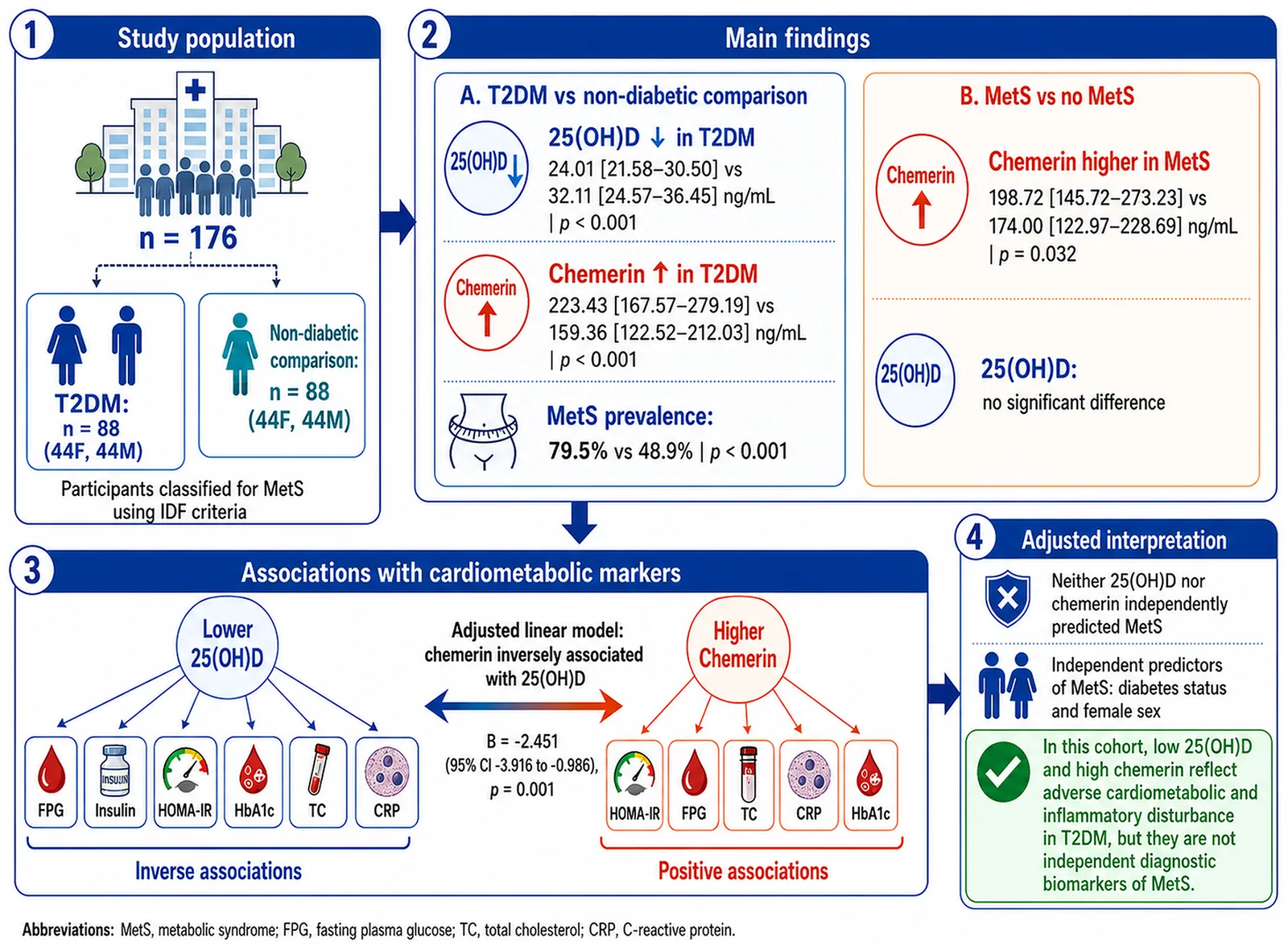

## 1 Introduction

The current and already significant prevalence of Type 2 Diabetes Mellitus (T2DM) presents a substantial global health burden to the community, including in the population of the Kingdom of Saudi Arabia (KSA) (1). T2DM is a complex form of metabolic disorder, which is usually intricately associated with the metabolic syndrome (MetS). Both environmental and genetic factors have been associated with the occurrence. Main features involved in the diagnosis of MetS include multiple metabolic abnormalities and prominent symptoms (2). Hallmark features of MetS include hypertension, dyslipidemia, low HDL cholesterol (HDL-C), and elevated levels of triglycerides (TG), central obesity, and hyperglycemia. Underlying mechanisms also include chronic inflammation (of low grade) and resistance to insulin (3).

The estimated global prevalence of DM is around 9.3% and is expected to rise to 10.2% by 2030 and 10.9% by 2045 (4). Within the Gulf region, the prevalence of T2DM has ranged from 8 to 22 %, with the greatest number of deaths reported in KSA (5).

With regard to the rising prevalence of MetS, even the international diabetes federation (IDF) emphasizes that around a quarter of the global population suffers from MetS (6). The prevalence of MetS has been reported to be about 12% in the United Arab Emirates (UAE). Among the Gulf Cooperation Council (GCC) countries, the prevalence of MetS ranges from 6% to 23.7% (7). MetS prevalence in KSA has been estimated at around 31.6% according to IDF guidelines, and 39.8% according to Adult Treatment Panel III (ATP III) standards (8).

Micronutrients, including vitamin D (VD), and adipokines, such as chemerin, have become significant players in the complex interplay among obesity, insulin resistance, and the pathogenesis of MetS and T2DM (9). In this study, VD status was evaluated using serum 25-hydroxyvitamin D [25(OH)D], because 25(OH)D is the major circulating form of VD. This form is widely used clinically and epidemiologically to classify VD deficiency, insufficiency, and sufficiency. VD receptors are expressed on pancreatic beta cells and in peripheral tissues responsive to insulin (10). This suggests that VD may play a role in glucose homeostasis. VD deficiency (VDD) has been associated with an increase in resistance to insulin, poorer control of glycemia, and adverse lipid profiles (11).

Chemerin is secreted by the liver and adipose tissue. It is implicated in conditions involving inflammation, adipogenesis, and metabolic regulation (12). Elevated circulating chemerin levels are linked to insulin resistance, obesity, dyslipidemia, and hypertension (13). Chemerin also promotes chronic, low-grade inflammation, a key characteristic of T2DM and MetS (14).

VDD and increased chemerin concentrations are independently linked to cardiometabolic risks and insulin resistance (15, 16). Chemerin generally promotes pro-inflammatory signaling pathways, whereas VD exerts anti-inflammatory effects. The interactions between these two molecules may influence the clustering and severity of MetS in individuals with T2DM (17). A study recently reported that among individuals with MetS, insulin resistance was associated with oxidative stress, adipokine dysregulation, and increased inflammation (18).

Although VD deficiency and elevated chemerin have each been linked to cardiometabolic risk, limited data are available on how serum 25(OH)D relates to chemerin and to the individual IDF-defined MetS components in individuals with T2DM, particularly in the Saudi population. The research gap addressed by this study is therefore not whether either marker alone diagnoses MetS, but whether the pattern of VD and chemerin concentrations is associated with MetS components, insulin resistance, inflammation, and dyslipidemia in T2DM. The specific MetS components investigated were central obesity/waist circumference, raised blood pressure, fasting plasma glucose (FPG), triglycerides, and HDL-C because these represent the diagnostic components in the IDF definition and are routinely measurable in clinical practice. Accordingly, the aim of this investigation was to explore associations of circulating 25(OH)D and chemerin with MetS components and related metabolic and inflammatory parameters in individuals with T2DM and in a non-diabetic comparison group.

## 2 Methods

This was a cross-sectional analytical study conducted among individuals with T2DM and non-diabetic comparison participants. Ethical approval for the present study was obtained from the King Abdulaziz University Regional Research and Ethics Committee (Ref. No. FMR-02-37-H). The study protocol was thoroughly explained to all participants, and written informed consent was obtained. The study was completed between August 2021 and December 2022. The research protocol was followed in accordance with local legislation, institutional requirements, and the Declaration of Helsinki.

Blood samples from all participants with diabetes were collected at the diabetic OPD of the primary healthcare center in Jeddah. The non-diabetic comparison group was recruited from the general population during the same study period through invitation and screening. The comparison group was defined by the absence of known diabetes and by FPG values below the diabetic range; therefore, the term “non-diabetic comparison group” is used. This group was not intended to be free of all cardiometabolic risk factors, and participants were therefore further classified according to the presence or absence of MetS using the same IDF criteria applied to the diabetic group. No financial incentive was offered to participants with T2DM or or to the non-diabetic comparison group. The complete histories of all participants were obtained, and those with known liver disease, chronic kidney disease, any inflammatory disease, or on current VD supplementation, or conditions expected to alter inflammatory or VD status were excluded. Participantswith T2DM were included in the diabetic group, and the non-diabetic comparison group was recruited separately for comparison. The researchers designed a questionnaire to collect sociodemographic data from all participants. The equal number of participants in the diabetic and non-diabetic groups and the equal sex distribution were predetermined by frequency matching to improve group comparability. Participants were enrolled consecutively until the planned numbers were achieved. Participants were classified as having MetS using the IDF definition for South/East Asian cut-off values “central obesity was mandatory, defined as waist circumference ≥90 cm in men or ≥80 cm in women, or assumed if BMI >30 kg/m^2^; MetS required central obesity plus any two of the following four components: triglycerides ≥1.7 mmol/L, low HDL-C (<1.03 mmol/L in men or <1.29 mmol/L in women), raised blood pressure (SBP ≥130 mmHg or DBP ≥85 mmHg or known hypertension), and raised fasting plasma glucose ≥5.6 mmol/L or known diabetes” (19). These criteria explain why some non-diabetic comparison participants could be classified as having MetS: MetS can be present in non-diabetic individuals when central obesity and at least two other diagnostic components are present, including impaired fasting glucose below the diabetes diagnostic range.

BMI was calculated as body weight (kg) divided by height in meters squared (BMI = kg/m^2^), and abdominal obesity was defined by waist circumference. HOMA-IR was calculated as “HOMA-IR = fasting glucose (mmol/L) × fasting insulin (μU/ml) / 22.5” (20). Because HOMA-IR is intended to estimate endogenous insulin resistance from fasting insulin and glucose, its interpretation may be affected by exogenous insulin therapy. Medication history was reviewed where available, and this issue has been clearly acknowledged as a limitation because detailed antidiabetic treatment data were not available for all participants. Therefore, HOMA-IR findings in treated T2DM participants should be interpreted cautiously.

For the categorization of VD, the Holick et al. (2007) classification was used. According to this classification, “Serum 25(OH)D level <=20 ng/ml, >20– <30 ng/ml, 30 ng/ml or more were classified as deficient, insufficient, and sufficient, respectively” (21).

Five milliliters of blood were collected from each participant after a 12-h overnight fast. Fasting plasma glucose (FPG), insulin, HbA1c, total cholesterol (TC), triglycerides (TG), LDL-C, HDL-C, C-reactive protein (CRP), chemerin, and 25(OH)D were measured. Serum CRP, chemerin, and 25(OH)D were analyzed by Enzyme-Linked Immunosorbent Assay (ELISA) using kits from Beijing Mesochem Technology Co., Ltd. (Beijing, China). According to the information provided in the kit booklet, the intra-assay and inter-assay variations were reported to be below 9 and 15%, respectively, for the chemerin and 25(OH)D assay kits. All laboratory measurements were performed according to the manufacturers' instructions and laboratory procedures. ELISA was used because it was the available validated laboratory platform for chemerin measurement in this research setting, and because chemerin is not routinely available on standard automated clinical chemistry or CLIA platforms in many clinical laboratories. TG, TC, HDL-C, LDL-C, FPG, and HbA1c were measured in the KAU hospital laboratory using automated analyzer kits (Roche Modular P-800, Germany).

### 2.1 Statistical analysis

The data were analyzed using SPSS v. 31. The normality of continuous variables was assessed using the Shapiro–Wilk/Kolmogorov–Smirnov test. As most variables were not normally distributed, the normality test results showed *p* < 0.05. Continuous variables were recorded as median [IQR] and compared using the Mann–Whitney *U*-test. Categorical ariables are reported as *n* (%) and compared by chi-square test or Fisher's exact test. Correlations between VD, chemerin, and various variables were computed using Spearman's rank correlation. Multivariable linear regression was used for VD and chemerin, and multivariable logistic regression was used for MetS status. Multiple outcomes were examined descriptively and analytically. Individual MetS components were not entered into the MetS logistic model because they define the outcome and would introduce circular reasoning. The multivariable models were used mainly to examine whether 25(OH)D and chemerin were independently associated with the main outcomes after adjustment for selected clinical covariates. 25(OH)D was modeled as a continuous variable in the regression analyses, while the deficient/insufficient/sufficient categories were used only for descriptive comparison. Multivariable linear regression was performed for continuous outcomes, specifically serum 25(OH)D and chemerin. The 25(OH)D model included diabetic status, MetS status, age, sex, BMI, HbA1c, CRP, and chemerin. The chemerin model included diabetic status, MetS status, age, sex, BMI, HbA1c, CRP, and 25(OH)D. BMI was included in the linear models because adiposity may confound both 25(OH)D and chemerin. For overall MetS status, multivariable logistic regression was used and odds ratios (ORs) with 95% confidence intervals (CIs) were reported. The logistic model included diabetic status, age, sex, HbA1c, 25(OH)D, and chemerin. BMI was not entered into the main MetS logistic model because central obesity/BMI is part of the IDF diagnostic definition of MetS, and including it could result in over-adjustment. Separate regression models were not run for diabetic and non-diabetic participants because of the modest sample size; instead, stratified Spearman correlations were reported for diabetic, non-diabetic, MetS, and no-MetS subgroups. Analyses were based on available complete data for each model. Because this was an exploratory study and several comparisons were performed, formal adjustment for multiple comparisons was not applied; exact *p*-values and 95% CIs are reported where applicable. Therefore, the regression findings should be interpreted as adjusted associations rather than causal effects.

## 3 Results

The study included 176 participants (88 diabetic and 88 non-diabetic comparison group, with 44 females and 44 males in each group). The diabetic subjects had significantly higher systolic (140.50 [135.00–150.00] vs. 130.00 [120.00–140.00] mmHg, *p* < 0.001), diastolic BP (85.00 [80.00–90.00] vs. 80.00 [75.00–85.00] mmHg, *p* < 0.001), BMI (29.73 [26.75–32.86] vs. 28.39 [25.97–31.19] kg/m^2^, *p* = 0.034), FPG, insulin, HOMA-IR, HbA1c, TC, TG, LDL-C, CRP, and chemerin (223.43 [167.57–279.19] vs. 159.36 [122.52–212.03] ng/ml, *p* < 0.001). They also had significantly lower HDL-C and 25(OH)D (24.01 [21.58–30.50] vs. 32.11 [24.57–36.45] ng/ml, *p* < 0.001) than the non-diabetic comparison group (Table 1). MetS was more common in diabetic participants than in non-diabetic comparison participants: 70/88 (79.5%) vs. 43/88 (48.9%) (*p* < 0.001). Chemerin was higher in participants with MetS than in those without MetS (198.72 [145.72–273.23] vs. 174.00 [122.97–228.69] ng/ml, *p* = 0.032), whereas 25(OH)D did not differ significantly between the MetS and no-MetS groups (25.93 [22.40–33.27] vs. 28.41 [23.83–35.28] ng/ml, *p* = 0.287) (Table 2).

**Table 1:** Comparison of general characteristics and biochemical values between T2DM and non-diabetic comparison groups (median [IQR]).

Variable	Non-diabetic comparison group	Diabetic group	*p*-value
Age (years)	53.00 [49.00–59.00]	55.00 [48.00–60.00]	0.539
Systolic BP (mmHg)	130.00 [120.00–140.00]	140.50 [135.00–150.00]	<0.001
Diastolic BP (mmHg)	80.00 [75.00–85.00]	85.00 [80.00–90.00]	<0.001
Height (m)	1.63 [1.57–1.69]	1.63 [1.59–1.66]	0.816
Weight (kg)	75.00 [66.75–88.50]	79.00 [70.00–90.00]	0.094
BMI (kg/m^2^)	28.39 [25.97–31.19]	29.73 [26.75–32.86]	0.034
Waist circumference (cm)	91.44 [85.72–101.60]	96.52 [88.27–104.14]	0.052
Hip circumference (inches)	39.20 [36.30–41.00]	40.00 [38.00–42.00]	0.115
FPG (mmol/L)	5.15 [4.92–5.26]	7.36 [6.60–8.75]	<0.001
Insulin (mIU/ml)	7.19 [6.10–10.12]	16.37 [13.90–20.07]	<0.001
HOMA-IR	1.66 [1.37–2.32]	5.56 [4.42–7.02]	<0.001
HbA1c (%)	5.02 [4.97–5.22]	7.90 [7.23–8.97]	<0.001
Total cholesterol (mmol/L)	2.61 [2.10–3.21]	4.56 [4.06–5.09]	<0.001
Triglycerides (mmol/L)	1.36 [1.03–1.67]	2.41 [1.75–2.70]	<0.001
HDL-C (mmol/L)	1.04 [0.80–1.19]	0.78 [0.60–1.00]	<0.001
LDL-C (mmol/L)	2.37 [2.11–2.98]	3.21 [2.60–3.57]	<0.001
CRP (mg/L)	0.56 [0.23–1.15]	4.62 [3.05–5.92]	<0.001
25(OH)D (ng/ml)	32.11 [24.57–36.45]	24.01 [21.58–30.50]	<0.001
Chemerin (ng/ml)	159.36 [122.52–212.03]	223.43 [167.57–279.19]	<0.001
**Categorical variables**	***n*** **(%)**	***n*** **(%)**	
Female	44 (50.0%)	44 (50.0%)	1.000
Male	44 (50.0%)	44 (50.0%)	
Hypertension: yes	21 (23.9%)	54 (61.4%)	<0.001
Hypertension: no	67 (76.1%)	34 (38.6%)	
Metabolic syndrome: yes	43 (48.9%)	70 (79.5%)	<0.001
Metabolic syndrome: no	45 (51.1%)	18 (20.5%)	

BMI, body mass index, FPG, fasting plasma glucose, HbA1c, glycated hemoglobin; HDL-C, high density lipoprotein-cholesterol; LDL-C, low density lipoprotein- cholesterol; CRP, C-reactive protein.

**Table 2:** Comparison of general characteristics and biochemical values by MetS status (median [IQR]).

Variable	MetS group	No MetS group	*p*-value
Age (years)	54.00 [48.00–60.00]	55.00 [50.00–59.50]	0.270
Systolic BP (mmHg)	140.00 [130.00–146.00]	130.00 [120.00–140.00]	<0.001
Diastolic BP (mmHg)	85.00 [80.00–90.00]	80.00 [75.00–84.00]	<0.001
Height (m)	1.63 [1.58–1.66]	1.63 [1.59–1.69]	0.329
Weight (kg)	82.00 [72.00–91.00]	70.00 [63.50–77.50]	<0.001
BMI (kg/m^2^)	30.49 [28.34–33.16]	26.75 [24.96–28.39]	<0.001
Waist circumference (cm)	96.52 [91.44–104.14]	86.36 [81.28–91.44]	<0.001
Hip circumference (inches)	40.00 [38.00–42.00]	39.00 [36.70–40.00]	0.005
FPG (mmol/L)	6.06 [5.18–7.83]	5.23 [5.14–5.87]	0.002
Insulin (mIU/mL)	14.05 [9.15–19.32]	9.63 [6.19–12.15]	<0.001
HOMA-IR	4.35 [2.15–6.29]	2.26 [1.45–3.67]	<0.001
HbA1c (%)	7.17 [5.20–8.20]	5.18 [4.99–6.60]	<0.001
Total cholesterol (mmol/L)	4.14 [2.95–4.84]	3.09 [2.23–3.82]	<0.001
Triglycerides (mmol/L)	1.81 [1.45–2.60]	1.56 [1.15–2.04]	0.001
HDL-C (mmol/L)	0.89 [0.64–1.09]	1.00 [0.74–1.16]	0.031
LDL-C (mmol/L)	3.03 [2.46–3.47]	2.39 [2.10–3.14]	<0.001
CRP (mg/L)	2.51 [1.07–5.35]	0.78 [0.36–2.86]	<0.001
25(OH)D (ng/mL)	25.93 [22.40–33.27]	28.41 [23.83–35.28]	0.287
Chemerin (ng/mL)	198.72 [145.72–273.23]	174.00 [122.97–228.69]	0.032
**Categorical variables**	***n*** **(%)**	***n*** **(%)**	
Non-diabetic comparison group	43 (38.1%)	45 (71.4%)	<0.001
Diabetic	70 (61.9%)	18 (28.6%)	
Female	67 (59.3%)	21 (33.3%)	0.002
male	46 (40.7%)	42 (66.7%)	
Hypertension: yes	56 (49.6%)	19 (30.2%)	0.019
Hypertension: no	57 (50.4%)	44 (69.8%)	

MetS, metabolic syndrome; BMI, body mass index, FPG, fasting plasma glucose, HDL-C, high density lipoprotein-cholesterol; LDL-C, low density lipoprotein- cholesterol; CRP, C-reactive protein.

Within the diabetic group, 18/88 (20.5%) had VDD and 47/88 (53.4%) had insufficient levels, whereas among non-diabetic comparison participants, 7/88 (8%) had deficiency and 32/88 (36.4%) had insufficient levels (*p* < 0.001). On the contrary, the distribution of VD status did not significantly differ by MetS status (*p* = 0.585) (Table 3).

**Table 3:** Serum 25(OH)D comparison between T2DM and non-diabetic comparison groups and metabolic syndrome (MetS) and no metabolic syndrome status.

Variable	Non-diabetic comparison group	Diabetic group	p-value
25(OH)D status in DM patients and non-diabetic comparison			
25(OH)D deficient	7 (8.0%)	18 (20.5%)	<0.001
25(OH)D insufficient	32 (36.4%)	47 (53.4%)	
25(OH)D sufficient	49 (55.7%)	23 (26.1%)	
25(OH)D status by metabolic syndrome status			
Vitamin D status by metabolic syndrome status			
	**No MetS**	**MetS**	
25(OH)D deficient	8 (12.7%)	17 (15.0%)	0.585
25(OH)D insufficient	26 (41.3%)	53 (46.9%)	
25(OH)D sufficient	29 (46.0%)	43 (38.1%)	

Higher FPG, insulin, HOMA-IR, HbA1c, TC, and CRP were all linked to lower VD. The strongest overall VD correlations were with TC (rho= −0.339), FPG (rho = −0.336), CRP (rho= −0.328), HOMA-IR (rho = −0.298), and HbA1c (rho = −0.288). These inverse relationships were evident in the MetS subgroup, and no correlation was found when the diabetic and non-diabetic comparison groups were examined separately (Table 4).

**Table 4:** Correlation of serum 25(OH)D with clinical and biochemical variables (spearman correlations).

Variable	Overall	Diabetic group	Non-diabetic comparison group	MetS group	No MetS group
Age (years)	−0.084 (0.268)	−0.012 (0.909)	−0.107 (0.320)	−0.135 (0.153)	0.044 (0.731)
BMI (kg/m^2^)	0.005 (0.944)	−0.037 (0.731)	0.191 (0.075)	0.006 (0.951)	0.138 (0.280)
Waist circumference (cm)	−0.054 (0.479)	−0.105 (0.332)	0.082 (0.447)	−0.093 (0.329)	0.057 (0.657)
Systolic BP (mmHg)	−0.073 (0.334)	0.041 (0.703)	0.144 (0.180)	−0.037 (0.696)	−0.096 (0.455)
Diastolic BP (mmHg)	−0.097 (0.201)	−0.076 (0.483)	0.067 (0.535)	−0.060 (0.529)	−0.173 (0.175)
FPG (mmol/L)	−0.336 (<0.001)	−0.106 (0.326)	0.062 (0.569)	−0.359 (<0.001)	−0.255 (0.044)
Insulin (mIU/ml)	−0.250 (<0.001)	0.067 (0.538)	0.141 (0.190)	−0.254 (0.007)	−0.165 (0.196)
HOMA-IR	−0.298 (<0.001)	−0.059 (0.585)	0.136 (0.207)	−0.324 (<0.001)	−0.185 (0.147)
HbA1c (%)	−0.288 (<0.001)	0.160 (0.136)	−0.026 (0.810)	−0.272 (0.004)	−0.205 (0.106)
Total cholesterol (mmol/L)	−0.339 (<0.001)	−0.066 (0.541)	−0.091 (0.401)	−0.364 (<0.001)	−0.244 (0.054)
Triglycerides (mmol/L)	−0.144 (0.057)	0.051 (0.636)	0.149 (0.167)	−0.143 (0.131)	−0.058 (0.653)
HDL-C (mmol/L)	0.147 (0.052)	0.041 (0.702)	0.017 (0.874)	0.124 (0.190)	0.129 (0.314)
LDL-C (mmol/L)	−0.086 (0.255)	0.052 (0.632)	0.144 (0.182)	−0.170 (0.071)	0.153 (0.231)
CRP (mg/L)	−0.328 (<0.001)	−0.188 (0.079)	0.070 (0.520)	−0.408 (<0.001)	−0.132 (0.302)

BMI, body mass index, FPG, fasting plasma glucose, HbA1c, glycated hemoglobin; HDL-C, high density lipoprotein-cholesterol; LDL-C, low density lipoprotein- cholesterol; CRP, C-reactive protein.

Chemerin was correlated with HOMA-IR (rho = 0.343), FPG (rho = 0.340), TC (rho = 0.334), CRP (rho = 0.310), and HbA1c (rho = 0.307). In the MetS subgroup, chemerin remained positively correlated with FPG, insulin, HOMA-IR, HbA1c, TC, and CRP (Table 5).

**Table 5:** Correlation of chemerin with clinical and biochemical variables (Spearman correlations).

Variable	Overall	Diabetic group	Non-diabetic comparison group	MetS group	No MetS group
Age (years)	−0.017 (0.828)	−0.058 (0.590)	0.002 (0.984)	−0.048 (0.611)	0.111 (0.385)
BMI (kg/m^2^)	0.057 (0.453)	0.198 (0.064)	−0.261 (0.014)	0.110 (0.247)	−0.332 (0.008)
Waist circumference (cm)	0.069 (0.366)	0.132 (0.221)	−0.062 (0.563)	0.041 (0.664)	−0.040 (0.754)
Systolic BP (mmHg)	0.057 (0.452)	−0.125 (0.247)	−0.052 (0.631)	0.017 (0.856)	0.022 (0.864)
Diastolic BP (mmHg)	0.072 (0.342)	0.062 (0.564)	−0.096 (0.373)	0.113 (0.232)	−0.107 (0.404)
FPG (mmol/L)	0.340 (<0.001)	0.258 (0.015)	−0.040 (0.709)	0.379 (<0.001)	0.222 (0.080)
Insulin (mIU/mL)	0.313 (<0.001)	0.050 (0.644)	0.043 (0.694)	0.285 (0.002)	0.218 (0.086)
HOMA-IR	0.343 (<0.001)	0.148 (0.168)	0.038 (0.728)	0.336 (<0.001)	0.243 (0.055)
HbA1c (%)	0.307 (<0.001)	0.115 (0.284)	−0.040 (0.710)	0.319 (<0.001)	0.225 (0.076)
Total cholesterol (mmol/L)	0.334 (<0.001)	0.035 (0.749)	0.144 (0.180)	0.263 (0.005)	0.396 (0.001)
Triglycerides (mmol/L)	0.194 (0.010)	−0.082 (0.449)	0.031 (0.777)	0.153 (0.105)	0.186 (0.145)
HDL-C (mmol/L)	−0.023 (0.764)	−0.047 (0.665)	0.254 (0.017)	−0.075 (0.431)	0.125 (0.327)
LDL-C (mmol/L)	0.160 (0.034)	0.119 (0.270)	−0.145 (0.177)	0.137 (0.149)	0.103 (0.423)
CRP (mg/L)	0.310 (<0.001)	0.024 (0.827)	0.084 (0.436)	0.335 (<0.001)	0.142 (0.269)

BMI, body mass index, FPG, fasting plasma glucose, HbA1c, glycated hemoglobin; HDL-C, high density lipoprotein-cholesterol; LDL-C, low density lipoprotein- cholesterol; CRP, C-reactive protein.

Regression analysis showed that diabetic status and female sex were independently associated with 25(OH)D status. In the adjusted chemerin model, VD was independently and inversely associated with chemerin (B −2.451, 95% CI −3.916–−0.986, *p* = 0.001) (Table 6).

**Table 6:** Associated variables with serum 25(OH)D, chemerin levels (multivariable regression models) and MetS status (logistic regression).

Variables	B	95% CI	*p*-value
Panel A. outcome: 25(OH)D (ng/ml)			
Diabetic group (vs. control)	−5.949	−10.347-−1.552	0.008
Metabolic syndrome (vs. no MetS)	0.225	−2.465–2.914	0.870
Age (years)	−0.000	−0.136–0.136	0.999
Female sex (vs. male)	2.154	0.105–4.202	0.039
BMI (kg/m^2^)	0.160	−0.111–0.431	0.247
HbA1c (%)	0.818	−0.228–1.864	0.125
CRP (mg/L)	−0.345	−0.924–0.234	0.243
Chemerin (ng/ml)	−0.028	−0.045-−0.010	0.002
Panel B. outcome: chemerin (ng/ml)			
Diabetic group (vs. non-diabetic comparison group)	4.535	−42.257–51.328	0.849
Metabolic syndrome (vs. no MetS)	14.504	−9.407–38.415	0.234
Age (years)	−0.055	−1.490–1.381	0.940
Female sex (vs. male)	−1.707	−23.376–19.962	0.877
BMI (kg/m^2^)	−0.382	−3.277–2.513	0.796
HbA1c (%)	8.028	−0.977–17.032	0.081
CRP (mg/L)	0.298	−7.091–7.687	0.937
25(OH)D (ng/ml)	−2.451	−3.916-−0.986	0.001
Panel C. Outcome: metabolic syndrome (logistic regression)			
Diabetic group (vs Non-diabetic comparison group)	6.885	1.690–28.057	0.007
Age (years)	0.960	0.917–1.006	0.084
Female sex (vs male)	3.266	1.599–6.672	0.001
HbA1c (%)	0.878	0.620–1.243	0.464
25(OH)D (ng/ml)	1.016	0.967–1.069	0.529
Chemerin (ng/ml)	1.003	0.998–1.008	0.289

BMI, body mass index, FPG, fasting plasma glucose, HbA1c, glycated hemoglobin; HDL-C, high density lipoprotein-cholesterol; LDL-C, low density lipoprotein- cholesterol; CRP, C-reactive protein.

In the logistic regression for MetS status, diabetes and female sex remained the only variables independently associated with MetS. Diabetic participants had nearly seven-fold higher odds of MetS compared with non-diabetic comparison participants (OR 6.885, 95% CI 1.690–28.057, *p* = 0.007), and females had about three times higher odds than males (OR 3.266, 95% CI 1.599–6.672, *p* = 0.001) (Table 6). Importantly, neither 25(OH)D nor chemerin was independently associated with MetS after adjustment, indicating that these markers should not be interpreted as independent diagnostic biomarkers of MetS in this cohort.

## 4 Discussion

The present study explored the association of serum 25(OH)D and chemerin with components of MetS and related metabolic and inflammatory parameters in individuals with T2DM and non-diabetic comparison participants. The main findings werethat participants with T2DM had lower 25(OH)D and higher chemerin than the comparison group, MetS was more frequent in the diabetic group, and chemerin was higher in participants with MetS. However, in the adjusted logistic regression model, neither 25(OH)D nor chemerin was independently associated with MetS. This point is important because it suggests that these markers are better interpreted as correlates of cardiometabolic disturbance in this cohort rather than as independent diagnostic biomarkers of MetS. No temporal sequence or causal pathway can be inferred from the present cross-sectional data. Our results support the findings of several other studies that found elevated chemerin (22–24) and a high prevalence of VDD in T2DM (25–27).

Serum 25(OH)D status in this Saudi population also needs to be interpreted in light of the main biological source of VD. In humans, the skin can synthesize cholecalciferol after exposure to ultraviolet B radiation, and 25(OH)D reflects VD derived from both cutaneous synthesis and intake. Therefore, low 25(OH)D in a sunny setting does not necessarily imply low sunlight availability. It may also reflect limited outdoor exposure, clothing practices, heat avoidance, darker skin pigmentation, sunscreen use, obesity-related sequestration, dietary intake, seasonality, or medication-related factors, and duration of diabetes and glycemic treatment. As the present study did not measure these factors, they remain possible sources of residual confounding.

Higher FPG, insulin, HOMA-IR, HbA1c, TC, and CRP levels were all linked to lower VD and these inverse relationships were also evident in the MetS subgroup. This finding suggests that 25(OH)D may be associated with glycemic control and metabolic homeostasis in this cohort, although causality cannot be inferred from this cross-sectional design. Some other studies have reported similar results (28–30).

The present study found that 25(OH)D was inversely correlated with FPG, insulin, HOMA-IR, HbA1c, TC, and CRP, whereas chemerin was positively correlated with HOMA-IR, FPG, TC, CRP, and HbA1c, further supporting their respective protective and adverse metabolic associations. These findings are consistent with previous reports showing that VDD is linked to insulin resistance and metabolic risk (31–33). Several studies reported that elevated chemerin is associated with MetS phenotypes, insulin resistance, and adverse lipid metabolism (34–36).

Similar to our study, Dahpy et al. reported substantially higher serum chemerin in MetS patients than in non-MetS individuals, and in diabetics than in non-diabetics and correlation and multiple linear regression analyses demonstrated that serum chemerin levels were significantly correlated with MetS and diabetes (37). Some other studies reported similar results (38–40). Higher levels of chemerin suggest the potential value of this adipokine as a research indicator in the pathophysiology of T2DM (41, 42).

The adjusted chemerin model showed an inverse association between VD and chemerin. However, this should be interpreted as an adjusted statistical association rather than evidence of a causal pathway. The association remained after inclusion of BMI in the model, which partially addresses adiposity as a confounder; nevertheless, residual confounding by adiposity distribution, dietary intake, sunlight exposure, physical activity, seasonality, and medication use cannot be excluded. The relationship between VD and chemerin may reflect shared links with adiposity, inflammation, insulin resistance, and lipid metabolism rather than a direct mechanistic axis. Low VD may coexist with metabolic dysfunction, while chemerin may reflect adipose-tissue-related inflammation and insulin resistance. Therefore, the present findings should be considered hypothesis-generating and confirmed in longitudinal or interventional studies designed to test directionality. Future studies should include detailed measurement of diabetes duration and medication exposure to improve interpretation of insulin resistance and biomarker associations.

We found positive correlations between serum chemerin and several metabolic parameters, including HOMA-IR, FPG, and TG, and negative correlations with HDL-C. A recent study also found elevated serum chemerin levels among T2DM participants compared to the control group and also reported its positive correlations with metabolic parameters (43). These findings suggest that chemerin may contribute to the dyslipidemia and impaired glucose homeostasis characteristic of T2DM (44). In contrast to our study findings, few other studies have reported no change or even a decrease in chemerin levels in individuals with T2DM. It highlights the complexity of chemerin's role and suggests potential influences of ethnicity, obesity status, and specific disease phenotypes (14, 45).

We found VDD and insufficiency in the majority of our diabetic group (20.5% deficient and 53.4% insufficient) compared with the non-diabetic comparison group (8% deficient and 36.4% insufficient). The present study's findings support the need to assess VD status in cardiometabolic research. Additionally, the fact that vitamin D status is strongly influenced by sun exposure, clothing practices, dietary intake, obesity, and season cannot be ignored. The present study observed VDD in individuals with MetS, though not statistically significantly, whereas chemerin levels were significantly higher in these subjects. MetS was significantly more frequent among diabetic than among non-diabetic comparison participants (79.5 vs. 48.9%) and among females than males (59.3 vs. 40.7%). In the present study, the decreased VD levels may reflect broader metabolic dysregulation rather than MetS status alone among the subjects. Higher chemerin levels suggest a more specific role for chemerin within the broader spectrum of MetS components, particularly regarding insulin resistance and related inflammatory markers (35, 46). It is also possible that VDD is a more general cardiometabolic risk marker and that its association with MetS might be confounded by other factors such as obesity, physical activity, seasonality, and background diabetes status.

Multivariable analysis showed that diabetes status and female sex, but not 25(OH)D or chemerin, were independently associated with MetS. Chemerin may still have research value as an inflammatory adipokine associated with metabolic disturbance, but its incremental clinical utility beyond routine measures such as FPG, HbA1c, CRP, and lipid profiles remains unproven. The current study's finding that MetS burden is greater in diabetes is expected, as T2DM and MetS share common mechanisms, including central adiposity, insulin resistance, hyperglycemia, dyslipidemia, and low-grade inflammation. These characteristics of participants with T2DM also support the greater burden of MetS in this cohort.

Our results demonstrated that female sex were independently associated with MetS in the adjusted logistic model, indicating that the difference between female and male sex was not due to chance. A plausible reason is that women in this cohort might have had higher adiposity and inflammatory abnormalities, which are regarded as some of the MetS-defining traits.

It appears that both VD and chemerin have complex relationships with glycemic parameters and lipid profiles, indicating that their relationships are both complex and interconnected in the context of the complicated pathophysiology of T2DM.

In the present study, chemerin showed positive correlations with most cardiometabolic risk factors, and 25(OH) showed negative associations with markers of metabolic dysfunction. These findings suggest that both markers may reflect broader metabolic and inflammatory disturbance. However, routine clinical decision-making in T2DM and MetS continues to rely primarily on established, lower-cost measures such as glycemia, HbA1c, lipid profile, blood pressure, waist circumference, and inflammatory markers when clinically indicated. Recently, Asghar et al., in their systematic review, reported that adipokine dysregulation is a key feature in individuals with MetS, who have higher levels of pro-inflammatory adipokines, including chemerin, which are strongly and positively correlated with IR and cardiometabolic risks (47).

Our study found altered levels of 25(OH) and chemerin in T2DM and MetS, but in logistic regression, neither was independently associated with MetS. This suggests that they should not be interpreted as causative factors but may be involved in metabolic dysregulation in these individuals. To elaborate on the exact mechanisms by which VD can regulate the expression or activity of chemerin, further research is needed, particularly since their associations with metabolic indicators of health are opposite.

**Figure 1:**
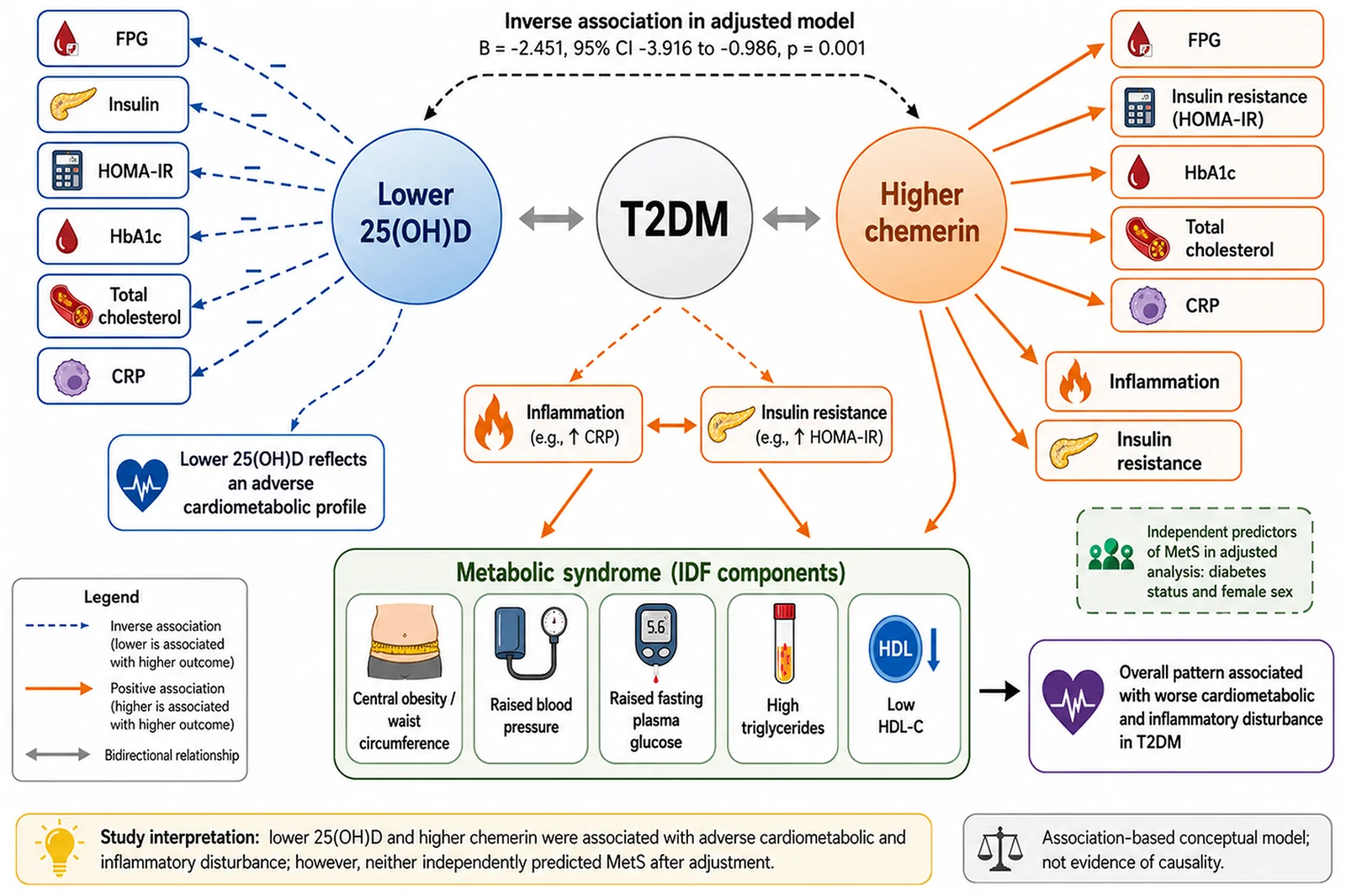
Conceptual relationship between 25(OH)D, chemerin, insulin resistance, inflammation, and metabolic syndrome components in type 2 diabetes mellitus.

The lower serum 25(OH)D and higher chemerin were associated with a less favorable cardiometabolic profile. Still, the adjusted models indicate that diabetes status and female sex, rather than 25(OH)D or chemerin, were independently associated with MetS. The findings therefore support further investigation of the VD-chemerin relationship as a research hypothesis rather than immediate clinical use as an independent MetS biomarker (Figure 1). These findings should be tested in larger longitudinal studies or intervention trials that more carefully measure sunlight exposure, dietary intake, physical activity, adiposity distribution, seasonality, and medication use.

## 5 Strengths of the study

Strengths of the study include equal numbers of participants with T2DM and non-diabetic comparison participants, equal sex distribution, use of standardized IDF criteria for MetS classification, comprehensive biochemical profiling, and regression modeling that included BMI as an adiposity-related confounder.

### 5.1 Implications of the study

The observed differences in VD and chemerin in people with T2DM suggest that these markers may help characterize metabolic and inflammatory disturbance in research settings. However, because neither marker independently was independently associated with MetS after adjustment, they should not be presented as stand-alone diagnostic biomarkers for MetS in clinical practice.

Future studies should examine whether measuring chemerin adds predictive value beyond standard clinical and biochemical panels, including fasting glucose, HbA1c, lipid profile, CRP, blood pressure, BMI, and waist circumference. Until such incremental utility is demonstrated, chemerin should be considered primarily a research biomarker rather than a routine clinical test.

Future research should also examine whether VD supplementation changes chemerin levels or improves metabolic outcomes in T2DM. Such studies should use longitudinal or randomized interventional designs and should include a detailed assessment of adiposity, sunlight exposure, dietary intake, physical activity, seasonality, and medication use.

Further studies with larger samples are needed to clarify temporal and causal relationships and evaluate the effectiveness of VD supplements in the long term on chemerin and other altered metabolic parameters.

### 5.2 Limitations

This study has some limitations that should be considered when interpreting the findings. First, its cross-sectional design allowed us to examine associations between 25(OH)D, chemerin, and MetS components, but it does not allow conclusions about causality or direction of effect. The study was also conducted at a single center and included a relatively modest sample of 176 participants. This limited the possibility of more detailed subgroup analyses and made separate adjusted regression models for diabetic and non-diabetic participants less feasible.

Although the equal number of diabetic and non-diabetic participants, as well as the equal sex distribution, improved comparability between groups, the findings may not be fully generalizable to other populations. Several potentially important confounders were not completely assessed, including sunlight exposure, outdoor activity, clothing practices, dietary VD intake, seasonal variation, detailed measures of adiposity, complete medication history, diabetes duration, and treatment regimen. This point is particularly important in the Saudi population, where cutaneous synthesis through sunlight exposure is expected to be a major source of VD. The absence of these data may have led to residual confounding in the observed associations with 25(OH)D.

Another limitation is related to the interpretation of HOMA-IR. Because exogenous insulin therapy can influence fasting insulin levels, incomplete information on antidiabetic medication use may have affected the interpretation of insulin resistance findings. In addition, no formal adjustment for multiple comparisons was applied, as this study was exploratory in nature. Therefore, the results should be interpreted with caution, focusing more on the overall pattern of associations rather than on individual *p*-values alone.

Larger multicenter studies, preferably with longitudinal or interventional designs, are needed to confirm these findings. Future work should also assess sunlight exposure, diet, physical activity, seasonality, medication use, and adiposity in greater detail to determine whether chemerin adds clinically useful information beyond established cardiometabolic risk markers.

## 6 Conclusion

In this cross-sectional Saudi cohort, participants with T2DM had lower serum 25(OH)D levels and higher chemerin concentrations than the non-diabetic comparison group. Chemerin levels were also higher among participants with MetS. However, after adjustment for relevant covariates, neither 25(OH)D nor chemerin independently associated with MetS. Instead, diabetes status and female sex emerged as the main independently associated variables. Overall, these findings indicate that 25(OH)D and chemerin are linked to cardiometabolic and inflammatory disturbances in this population, but they should not be regarded as causal factors or independent diagnostic biomarkers of MetS. Larger longitudinal and interventional studies are needed to clarify the direction of these associations and determine their possible clinical value.

## Data Availability

The raw data supporting the conclusions of this article will be made available by the authors, without undue reservation.
